# EMI-LTI: An enhanced integrated model for lung tumor identification using Gabor filter and ROI

**DOI:** 10.1016/j.mex.2025.103247

**Published:** 2025-02-27

**Authors:** Jayapradha J, Su-Cheng Haw, Naveen Palanichamy, Kok-Why Ng, Muskan Aneja, Ammar Taiyab

**Affiliations:** a Department of Computing Technologies, School of Computing, SRM Institute of Science and Technology, Kattankulathur, Tamil Nadu, 603203, India; b Faculty of Computing and Informatics, Multimedia University, Jalan Multimedia, 63100, Cyberjaya, Malaysia

**Keywords:** Lung cancer, Convolutional neural network, CT images, Region of interest and data augmentation, Convolutional Neural Network, Region of Interest, Gabor Filter, Data Augmentation)

## Abstract

In this work, the CT scans images of lung cancer patients are analysed to diagnose the disease at its early stage. The images are pre-processed using a series of steps such as the Gabor filter, contours to label the region of interest (ROI), increasing the sharpening and cropping of the image. Data augmentation is employed on the pre-processed images using two proposed architectures, namely (1) Convolutional Neural Network (CNN) and (2) Enhanced Integrated model for Lung Tumor Identification (EIM-LTI).

• In this study, comparisons are made on non-pre-processed data, Haar and Gabor filters in CNN and the EIM-LTI models. The performance of the CNN and EIM-LTI models is evaluated through metrics such as precision, sensitivity, F1-score, specificity, training and validation accuracy.
• The EIM-LTI model's training accuracy is 2.67 % higher than CNN, while its validation accuracy is 2.7 % higher. Additionally, the EIM-LTI model's validation loss is 0.0333 higher than CNN's.
• In this study, a comparative analysis of model accuracies for lung cancer detection is performed. Cross-validation with 5 folds achieves an accuracy of 98.27 %, and the model was evaluated on unseen data and resulted in 92 % accuracy.

## Graphical abstract

**Figure:**
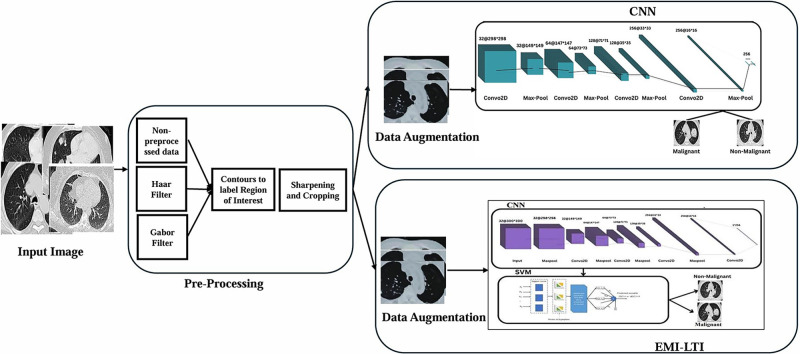

Specifications table

**Table:** 

Subject area:	Computer Science
More specific subject area:	Deep Learning
Name of your method:	Convolutional Neural Network, Region of Interest, Gabor Filter, Data Augmentation)
Name and reference of original method:	Mahmood, S.A., Ahmed, H.A. An improved CNN-based architecture for automatic lung nodule classification. Medical and Biological Engineering & Computing 60, 1977–1986 (2022).
Resource availability:	https://doi.org/10.1007/s11517–022–02578–0]

## Background

In today's world, cancer is a major life-threatening disease and one in five individuals are affected by cancer [1]. Lung cancer is shown as a pulmonary nodule, thus identifying the size of the nodules may be used to diagnose the cancer as beginning stage or malignant stage. By measuring the nodules, the cancer can be identified and treated as early as possible. Lung cancer is of two types small cell lung cancer and non-small cell lung cancer [2]. Medical images can be captured using traditional image techniques such as magnetic resonance imaging (MRI) and computed tomography (CT). The images from CT and MRI are widely used in various image acquirement techniques, however, each possesses different characteristics and purposes. CT scan captures the inner parts of the body using X-rays. The CT scan images can be widely used to identify the anomalies in the lungs [3]. However, the MRI captures the inner body images using radio and powerful magnetic waves. MRI results in extreme-resolution images from various parts of the body such as the brain and spine [4]. CT image segments are easy to capture and are non-offensive and simple. As mathematical methodologies and machine learning are rapidly developing, CT images are extensively used as an input in cancer detection [5]. In clinical images, 2D and 3D images are used to categorize lung cancer. Recent research studies focused on ML algorithms and deep learning (DL) algorithms for the classification of images [6]. There are various DL models, but the most widely used model for image classification is Convolutional Neural Network (CNN) [7]. CNN is remarkably used because of its hierarchical data representation and automatic pattern learning from the input images. The above reasons make CNN perform efficiently to classify images even if the input images are substantially different from the trained samples, though they carry an identical class of features [8].

For the detection of lung cancer, the captured raw images cannot be fed directly into the ML models, as the preprocessing of the images is important. The detection accuracy of lung cancer detection will fall without pre-processing the raw CT images. As a result, preprocessing is mandatory for various classifier combinations to produce good accuracy [9]. Consequently, the extraction of the lung CT image segmentation is an essential part of pre-processing [10]. Feature extraction plays a major role in the pre-processing stage as it is used to identify and extract the various portions of the images. Our research has focused on the preprocessing of clinical images to give the best performance. To detect the various features of the images clearly, image enhancement is needed. In our research work, the following steps are carried out as the pre-processing steps (i) Gabor filter is used for image enhancement, (ii) Lung ROI segmentation using contours and (iii) Enhancement of image sharpness and (iv) Pre-processed images are fed into models.

### Machine learning algorithms in lung cancer detection

Identification of lung cancer by applying CT scan images is innovative, especially because the mortality caused by lung cancer is high [11]. To overcome these limitations [12], computer-aided automatic diagnosis methods using image processing techniques and Artificial intelligence paradigms like Artificial Neural Networks (ANNs) as well as Support vector machines (SVMs) have been settled to increase the efficiency of diagnosis of lung cancer in reducing human interferences. Notably, despite the supportive role that CT scans play in the detection of lung cancer [13], the high false-positive results whose advancement led to the creation of the minimally invasive multimodality image-guided (MIMIG) interventional system are studied. This comprises CT image segmentation, electromagnetic tracking as well as molecular imaging to improve early diagnosis and treatment. However, in Lung Cancer detection, the accuracy of using 3D convolutional neural networks CNNs detection has been noted to be high the precision and recall rates are also high [14]. Thus, the utilization of deep learning techniques for the detection of lung cancer using CT scan images is gradually increasing. The work [15] has found that using automation in the analysis of chest CT scans is useful in enhancing lung cancer identification reliability and speed, which is important in the patient's success. The author also employed the ensemble classifier and applied the principal component analysis (PCA) on the Elvira Biomedical Dataset specifically for lung cancer prediction. It most probably deals with the performance of different classifiers including RF, VM, KNN, and NB in terms of lung cancer possibility. Thus, by applying ensemble methods and dimensionality reduction with the help of the PCA method, the work contributed to improving lung cancer prediction models in terms of both accuracy and time. Jallal et.al [16] discussed using the KNearest Neighbors (KNN) algorithm for predicting lung cancer. The findings of the study also indicate that machine learning algorithms, inclusive of KNN, can help increase the efficiency of early diagnosis and treatment of lung cancer. A model was proposed by [17], for employing supervised learning algorithms to identify Lung Carcinoma. Techniques like logistic regression, a Support Vector Machine (SVM), and KNN have been used to detect and categorize lung carcinoma cases.

Lung image enhancement [18] using the Gabor Filter and smoothing was done using the Gaussian Filter to boost the prediction accuracy of classifiers usable to identify the right diagnosis namely Support Vector Machine and Fuzzy C-Mean Clustering. Genç.al [19] aimed to identify the best filtering techniques, including the Butterworth filter, Gaussian filter, Gabor filter, fast Fourier filter, and Discrete wavelet transform, especially in the detection of lung cancer from CT images. Moreover [20], applied the Gabor filter and Histogram Equalization+CLAHE filter on the original datasets were applied to detect lung cancer cells and stressed the role of the filter in the preprocessing step for accurate classification. Further [21], described an algorithm for lung cancer detection that includes median filtering for image smoothing, contrast adjustment for image enhancement, segmentation using morphological watershed operations, and Otsu's thresholding. This gives credence to filtering techniques when it comes to the preprocessing stage. Altogether, these investigations shed light on the fact that it is essential to employ appropriate filter techniques to make advancements in lung cancer dataset pre-processing with the highest probability of classification accuracy and the least execution time required. By integrating the filtering methods, the researchers endeavor to achieve high-quality data for the subsequent machine learning methods to enhance lung cancer's early diagnosis and differentiation.

## Method details

### Theoretical framework and proposed prototype

#### Proposed lung nodule identification and classification

Early detection of the lung nodules reduces the patient's death rate. The biggest problem with cancer detection techniques is the false positive results, which lead to inaccurate identification. An automatic lung nodule detection model has been proposed to eradicate the inaccurate identification of lung cancer. Two models were proposed and compared in this paper. The first proposed model is CNN and the second is the Enhanced Integrated Model for Lung Tumor Identification (EIM-LTI). The proposed models comprise of (i) Pre-Processing and Segmentation, (ii) Enhancing the sharpness and cropping image, (iii) Data Augmentation and (iv) Classification. However, the proposed CNN and EIM-LTI follow the same procedure for pre-processing, segmentation, enhancing the sharpness and cropping the images, the data augmentation and classification of the model differs. Primarily, the dataset is pre-processed using the Gabor filter and then the images in the dataset are segmented by the Region of Interest (ROI) using contours. This paper proposes a hybrid of ROI using contours with a Gabor filter for pre-processing, which greatly helps in classification.

#### Pre-Processing and segmentation

Image pre-processing is required to improve the quality of the lung images as the deprived-quality images can reduce the accuracy of the identification of the lung nodules

#### Implementation of Gabor filter

The Gabor filter is applied to improve the quality of the image. Gabor filters are linear frequency and orientation-selective filters [22]. The Gabor filter improves the receptivity difference between the nodules and the normal regions. Thus, the Gabor filter plays a major role in the image pre-processing. The proposed methodology uses the mathematical equation as shown in Eq. (1).

(1) $$filt_{img}=np.abs(gab\left(gry_{img,\;}freq=freq,\;theta=theta,\;sig_{x}=sig_{x},\;sig_{y}=sig_{y}\right)$$

The parameters for the Gabor filter are freq = 5, $sig_{x}$=0.25 and $\text{si}g_{y}=0.25$.

*The filtered image is normalized as shown in* Eq. (2) *.*

(2) $$filt_{img}=\left(filt_{img}-np.min_{\left(filt_{img}\right)}\right)/\left(np.max_{\left(filt_{img}\right)}-np.min_{\left(filt_{img}\right)}\right)*255$$

**Fig. 1:**
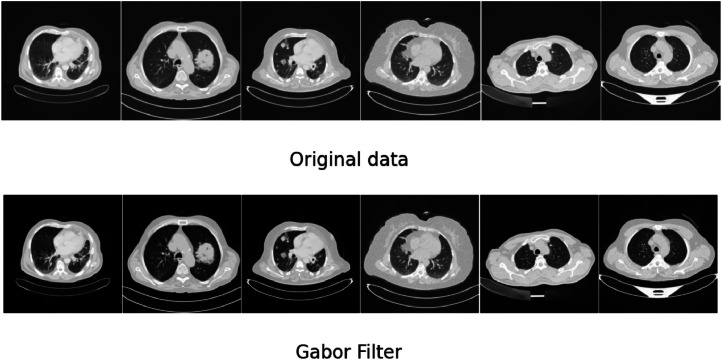
Gabor filter Raw data and Gabor filtered data.

Where the normalized images are converted into uint8 images. Fig. 1 shows the output of Gabor filter on the raw data.

**Fig. 2:**
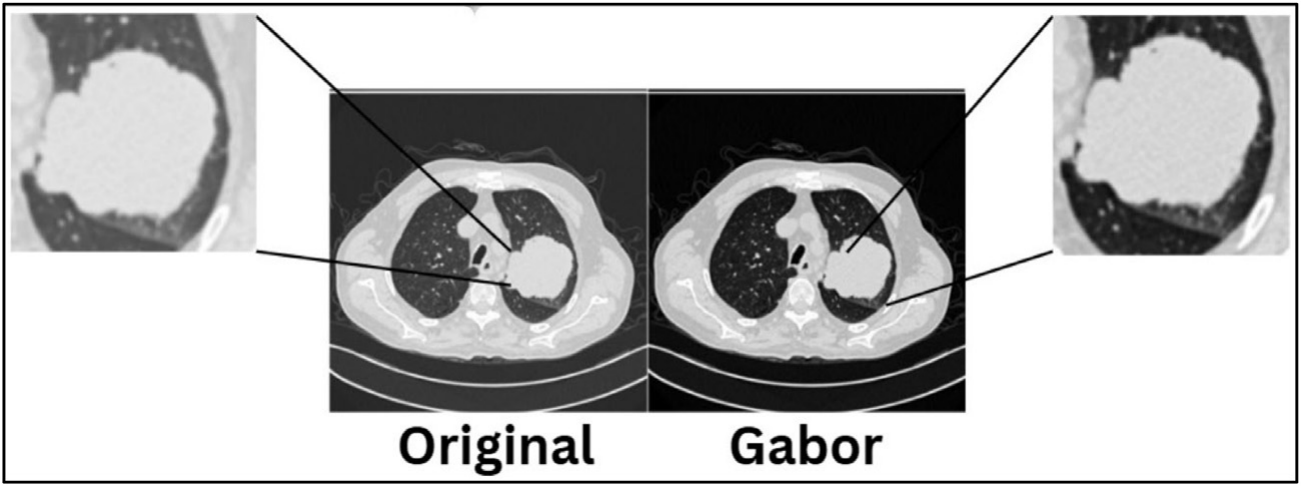
Representation of difference the Gabor filter created.

Though Fig. 1 depicts the raw data and Gabor filtered data, the differences are very mild. In order to represent the difference the Gabor filter creates, Fig. 2 is showcased.

#### Lung segmentation using contours to label region of interest (ROI)

**Fig. 3:**
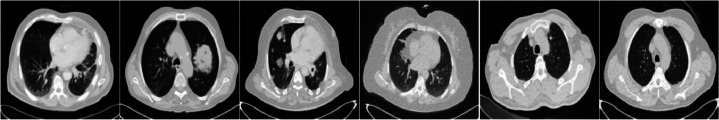
ROI extraction using contours.

After applying the Gabor filter, the function is to find the lungs and crop the lung region. Usage of contours to label ROI [23] is done for efficient data processing and helps focus the specific region of the image analysis. Contours help recognize and border the edges of the object in the interior of an image, thus, only the relevant images are analyzed and processed, which can substantially accelerate the image processing. Contours also reduce the noise and improve the edges in the ROI's interior, making the image finer and more identified. The following steps extract the images: i) converting the image to a grayscale, ii) applying the adaptive thresholding to create a binary image, iii) finding contours of lung bright areas, iv) finding the bounding box for the largest contour, v) adding padding to the bounding box, vi) Ensuring the coordinates are within the image bounds and vii) cropping the lung region. After the ROI part is extracted, it is sharpened and cropped further for classification purposes. Fig. 3 depicts the ROI extraction using contours.

#### Increasing sharpening and cropping image

Though the contours to label ROI were implemented, the images need to be clearer and more defined for further *classification.* Thus, the sharpening kernel [24,25] is implemented in the segmented image further to improve the features and edges of the images and make the image clearer and more defined. A sharpening kernel is used to excerpt certain features from the images. The sharpening kernel as per Eq. (3) is multiplied with the input images to give a clearer image as shown in Eq. (4).

(3) $$Sharp_{kernel}=\left[\begin{array}{ccc} -1 & -1 & -1\\ -1 & 9 & -1\\ -1 & -1 & -1 \end{array}\right]$$

(4) $$sharp_{image}=filter\left(input_{img},-1,Sharp_{kernel}\right)$$

**Fig. 4:**
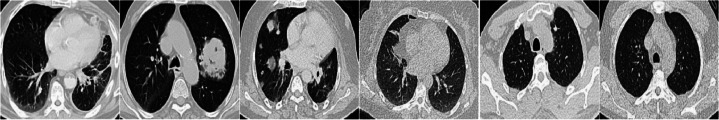
Sharpening and cropping.

**Fig. 5:**
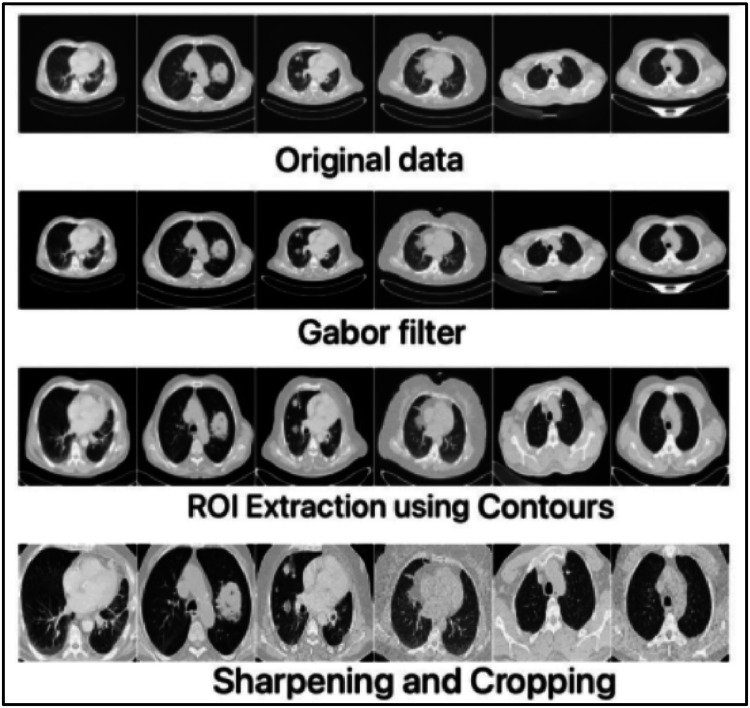
Outputs of the entire preprocessing.

The sharpened images are further cropped and respective malignant and non-malignant images are loaded into the respective folder for classification. Fig. 4 depicts the result of the sharpening and cropping of the images, while Fig. 5 depicts the overall output of the pre-processing.

#### Working process of the proposed convolutional neural network

##### Data augmentation

**Fig. 6:**
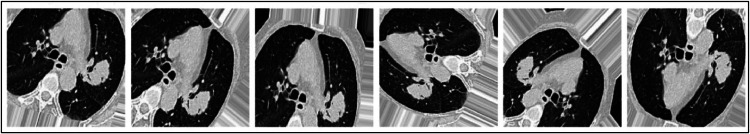
Images generated after data augmentation.

In deep Learning (DL), data augmentation is implemented to generate samples from the persisting training images. Many data augmentation techniques such as scaling, rotation and contrast alteration, are available to generate samples of images to increase the accuracy of the proposed methodology [26]. The number of augmented images generated using Datagen cannot be precisely counted because data augmentation is applied dynamically during training, creating unique transformations of each image on the fly. This means the same image can generate different variations across batches and epochs, leading to potentially infinite unique augmented images throughout the training process. The proposed work has set various parameters for the artificial image generator. Table 1 shows the values set for the image generator. Fig. 6 shows the series of images generated after data augmentation.

**Table 1:** Set value for the image generator parameters.

Parameters	Set value
rescale	1./255
rotation_range	20
width_shift_range	0.1
height_shift_range	0.1
fill_mode	`reflect'
validation_split	0.2
zoom_range	10 %
horizontal_flip	True

CNN is the primary element of the artificial neural network that handles data in a grid topology [27]. Various convolutional layers have been implemented to learn multiple dimensions of the input data automatically and flexibly. CNN is defined as the multilayer deep learning architecture utilized in image processing. It takes images as inputs and forbids and categorizes all the characteristics in the images with different processes. These images first go through the convolutional, pooling and fully connected layers. Thus, CNN has become more effective in pattern, image recognition, classification and segmentation. In our work, after pre-processing the dataset, the CNN architecture has been developed based on the IQ-OTH/NCCD dataset in Python 3 [28]. The CNN architecture implemented in our study is explained as follows.

###### Input layer

The architecture is initiated with the input layer, where the image is accepted as a 300 * 300(width* height) image; however, it does not perform any computations at this level.

###### Convolutional layer (3 × 3)

There are four convolutional (conv) layers in the proposed CNN architecture. The first conv layer performs 32 filters with 3 * 3 dimensions to the input image. On the spatial hierarchies and patterns, each filter glides over the image. This layer is handy in identifying details such as the edges and other textures within the image. The second conv layer uses a filter of 64 sizes by 3 * 3. This layer then recognizes more complex features than those identified in the first layer but includes them in some features. The third conv layer is of 3 * 3 filters with 128 numbers. This layer extends dig-out features but in a more complicated manner enduring on the classified feature abstraction method. The last conv layer, the fourth one, uses 256 filters of size 3 × 3. By the last layer, many detailed features of the input image may be obscured by higher-order features.

###### Max pooling layer (2 × 2)

Completing the initial layers, there is the first conv layer, after which a max pooling layer with a pool size of 2 × 2 comes. This layer performs subsampling by taking the maximum value of every 2 × 2 window and shedding the spatial dimensions down by half. This is useful in making the network more invulnerable to variations and fluctuations in the position of features as well as cost savings. Further, for each conv layer, the max pooling layer decreases the spatial extent of the feature maps.

###### Flatten

After, conv and pooling layers, the structure is flattened to have one dimension. This is done in a bid to prepare it to be fed to the subsequent fully connected layers.

Dense (512 neurons, ReLU): The first fully connected layer has 512 neurons, the activation function used is called ReLU which stands for Rectified Linear Unit. This layer learns the features acquired by the conv layers and then is used to classify the image. The ReLu function of the dense layer is as shown in Eq. (5).

(5) ReLu(y)=maximum(0,y)

**Fig. 7:**
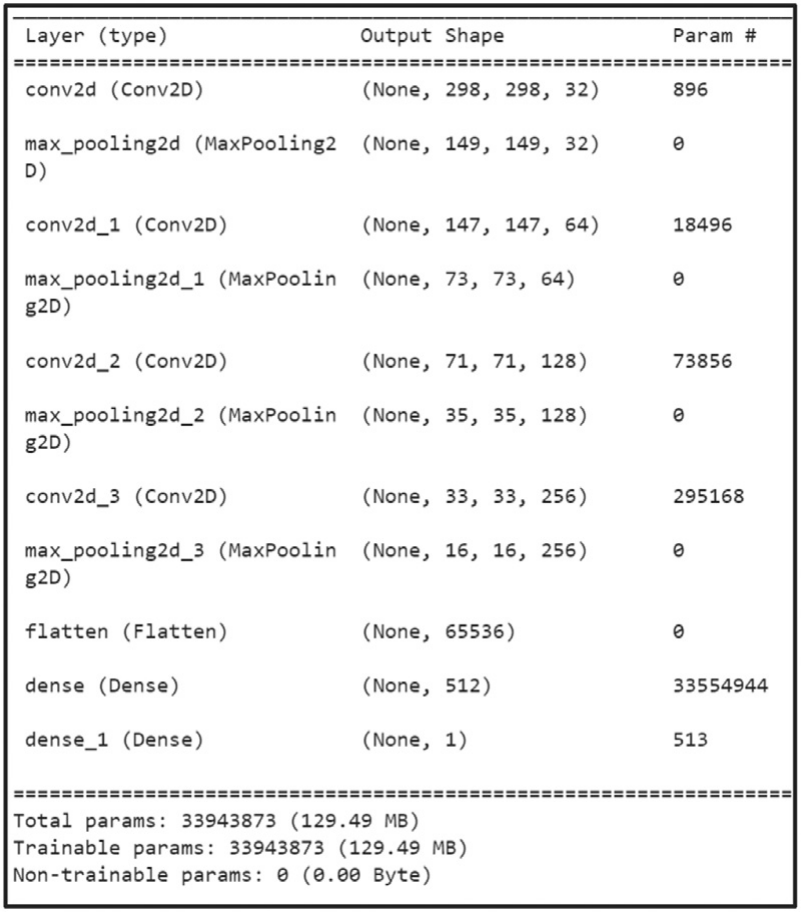
CNN model summary.

Dense (1 neuron, Sigmoid): The final layer of the neural network has one neuron and a hyperbolic activation function is used in the final neuron. The sigmoid function scales down the calculated value, giving it a probability form between 0 and 1 useful for binary classification. The sigmoid function of the dense layer is shown as in Eq. (6) and the hyperparameters of the proposed CNN model are shown in Table 2. The summary of the implemented CNN model is shown in Fig. 7.

(6) $$\sigma \left(y\right)=\;1\;/\;1+e^{-y}$$

**Table 2:** Hyper-parameters of designed CNN model.

Parameters	Value
Input Size	300×300
Batch Size	64
Learning Rate	0.0001
Optimizer	ADAM
Activation Function	ReLu, Sigmoid
Loss Function	Binary Cross Entropy

**Fig. 8:**
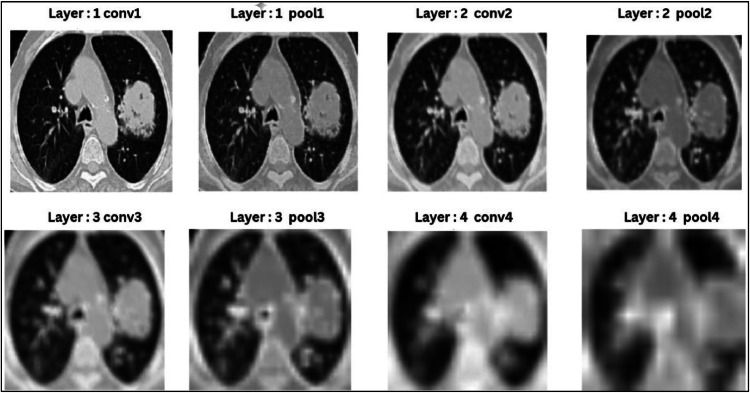
Output of the CNN model.

**Fig. 9:**
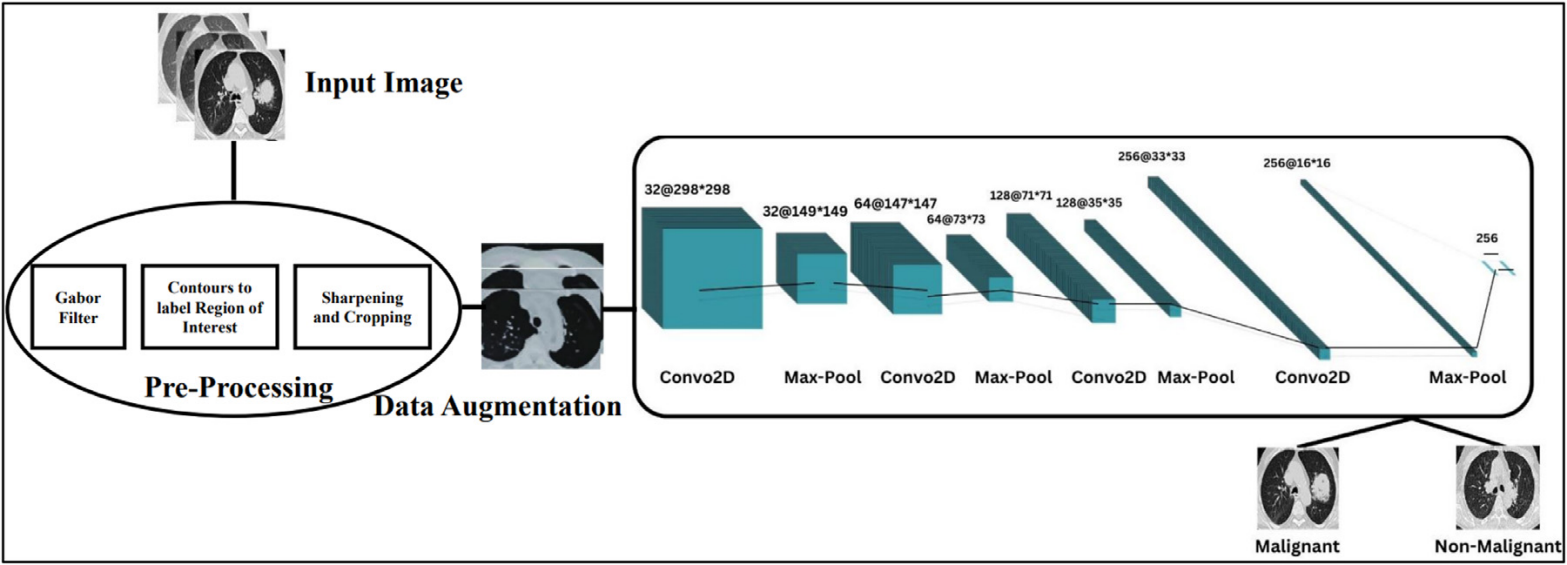
Architecture diagram of the Proposed CNN Model.

Fig. 8 depicts the output images of the CNN model. Layer 1: Conv1 + Pool1 retains most of the original image detail while emphasizing edges and textures. Pooling slightly reduces resolution, focusing on prominent features. Layer 2 Conv2 + Pool2 begins losing finer details as it combines edges into patterns and pooling reduces the image size, discarding minor variations while retaining critical patterns. Layer 3: Conv3 + Pool3 abstracts shapes and regions, losing specific textures and pooling further reduces spatial resolution, keeping only prominent structures. Finally layer 4: Conv4 + Pool4 becomes highly abstract, losing most fine details to focus on task-specific, high-level features critical for classification. As the layer moves on, the size of the images get reduced, thus all the layer images have been rescaled from their original size in the CNN layers to Visualize. The complete architecture of the proposed model is shown in Fig. 9.

#### Working process of the proposed EIM-LTI

##### Data augmentation

**Fig. 10:**
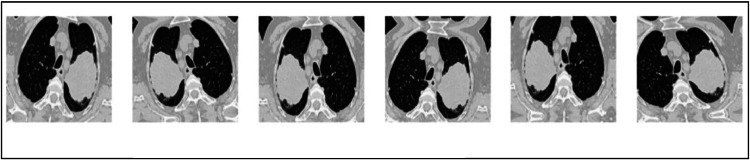
Subset of new images after augmentation.

In EIM-LTI, the images are augmented using techniques such as 1) rescaling, 2) rotation range, 3) shift ranges, 4) fill mode, 5) validation split and 6) horizontal flip to increase the accuracy of the proposed methodology. In the proposed EIM-LTI, various parameters have been fixed for the image generator. Table 3 shows the values set for the image generator and Fig. 10 shows the series of images generated after data augmentation. The rotation_range of the EIM-LTI is varied for data augmentation by 10°.

**Table 3:** Set value for the image generator parameters.

Parameters	Set value
rescale	1./255
rotation_range	10
width_shift_range	0.1
height_shift_range	0.1
fill_mode	`reflect'
validation_split	0.2
zoom_range	10 %
horizontal_flip	True

In the proposed EIM-LTI model, the hybrid of CNN and SVM are implemented to overcome the limitations in CNN. Though CNN has various features such as (1) Local Sensitivity, (2) Parameter Sharing, (3) Hierarchical Feature Learning and (4) Elevated Precision. However, the CNN possesses the above advantages, it also has a few disadvantages such as (1) CNNs may entail high computational cost and may need a lot of computational power, particularly for deep CNNs and (2) if not well regularized, CNNs are prone to memorizing a training set and hence will have poor performance on new data. To overcome the disadvantages of complexity and overfitting, an enhanced hybrid model of CNN+SVM is proposed to classify lung cancer images. The CNN+SVM hybrid model integrates the extension of CNN and the classification ability of SVMs. CNN is employed to extract high-level features from the input, which are then fed to an SVM for classification. The primary purpose of proposing the EIM-LTI model is to possess the following advantages: (i) The capacity to work with samples of large dimensions, (ii) Support Vector Machines (SVM) has a unique luxury over other learning algorithms with regards to robustness and generalization. (iii) Interpretability: SVMs give a sleek decision boundary and during classification, their working can be easily understood, thus, the model is more transparent. (iv) Handling Imbalanced Data: Using feature vectors of samples, the SVMs can be trained with less attention paid to imbalanced sample counts, but by tuning class weights, it can be useful in some cases. (v) Multi-Class Classification: SVMs are ideal for use in complex classifications because they work in multi-class settings as a matter of course [29].

The proposed EIM-LTI model follows the same pre-processing steps such as (1) Implementation of the Gabor Filter, (2) Segmentation using contours to label ROI and (3) Enhancing the sharpness and cropping image. After the pre-processing steps, EIM-LTI model varies in the data augmentation and the classification process of the images. The enhanced hybrid model has implemented the CNN model and the output of the CNN model is fed into the SVM model. The architecture of the CNN model is almost like the CNN model we implemented above, with a few changes in the feature map extraction. Additionally, two features are introduced in the hybrid model such as (1) kernel regularization and (2) dropout. The main purpose of adding the above two features is to prevent the model from being overfit. The first fully connected layer has 512 neurons and uses the ReLU activation function for non-linearity owing to its higher number of neurons; L2 regularization with a factor of 0.01 is also applied to prevent overfitting. A drop out layer with parameter 0.5 is included to remove 50 % of the neurons during training randomly and thus will reduce the problem of overfitting as shown in Eq. (7). The final dense layer consists of one neuron with the sigmoid activation function appropriate for binary classification.

(7) $$Dense_{layer}=\left(512,\;act_{fnc}\left(relu\right),\;kernel_{regulizer}=l2\left(0.01\right),\;Drop_{out}=0.5\right)$$

The proposed model employs the Adam optimizer, one of the fastest stochastic optimization techniques. So, the loss is set binary cross entropy which is suitable for binary classification since the output is a probability value. The parameter of the model restore_best_weights=True allows it to be provided with the best-observed weights according to the validation loss after the training is over and the model is finally evaluated.

The working of the SVM comprises three following steps, (1) Feature Extraction, (2) Feature Generation and (3) SVM Training that trains SVM classifier using the extracted features and the labels associated with the features. This approach is built on the high feature extraction ability of the CNNs and the accurate classification ability of the SVMs. In this case, the feature vector is extracted with the help of the CNN and then fed to the SVM. The created CNN model is provided as input to the SVM model and a new model is developed using the model class of Kera's. This model accepts the same input as the first CNN model but returns the features decoded from the CNN model's second last layer, mod. Layer [−2].out as shown in Eq. (8).

(8) $$feature_{extrac}=Mod\left(inp=mod.inp,out=mod.layer\left[-2\right].out\right]$$

This is usually set as the last hidden layer with many neurons, that distinguishes more general features of the input data. The $\text{featur}e_{\text{extrac}}\;$model used in this study is used to extract features from the training features ($\text{trai}n_{\text{data}})$ as well as validation features ($\text{va}l_{\text{data}})$. The prediction method produces feature vectors for samples in the datasets as shown in Eqs. (9) and (10).

(9) $$train_{data}=\;feature_{extract}.pred\left(a_{train}\right)$$

(10) $$val_{data}=\;feature_{extract}.pred\left(a_{val}\right)$$

**Fig. 11:**
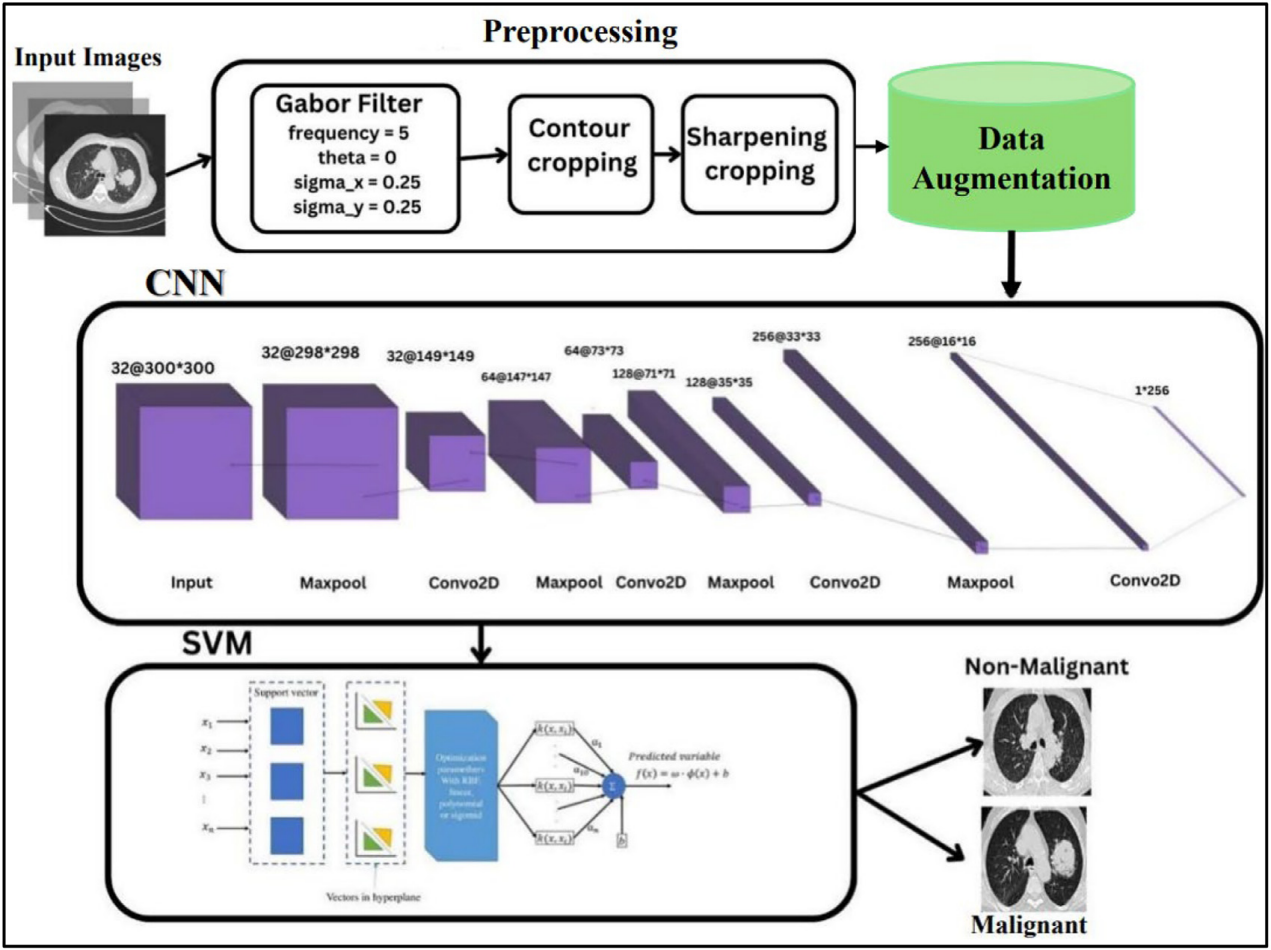
Architecture of the proposed EIM-LTI model.



An SVM classifier is created using the SVC class from scikit learn wherein different classifiers require different types of kernels with different parameters. The cut-off option is kept as the default ‘kernel=linear’ meaning that linear kernel is to be applied as shown in Eq. (11). The training of the SVM model is processed using the extracted features of images and the flattened vectors of training labels, respectively. The ravel method ensures that trained labels are a 1D array as shown in Eq. (12) because the SVM classifier expects it in this form. Finally, the SVM model is predicted based on the training data. The architecture of the EIM-LTI model is depicted as shown in Fig. 11.

(11) $$SVM_{mod}=\;SVC\left(kernel_{linear}\right)$$

(12) $$SVM_{mod\left(fit\right)}=\;train_{data},\;b_{train}\left(ravel\right)$$

## Method validation

### Dataset

The experiment was implemented using Python 3 with Windows OS. The dataset used in the experimental work is “The IQ-OTH/NCCD lung cancer”. The dataset can be found at https://www.kaggle.com/datasets/hamdallak/the-iqothnccd-lung-cancer-dataset?-Resource=download. The dataset comprises lung cancer CT scans from Iraqi hospitals. The dataset consists of 1190 images with 140 different cases and the images are categorized into three different types such as 1) Normal, 2) Benign and 3) Malignant. As the images of benign and normal are very less, they are merged and two classes such as 1) malignant and 2) non-malignant, were made for the classification of lung cancer. The 80:20 ratio is considered for the training and learning process. The results were evaluated for both CNN and the EIM-LTI model using the metrics (1) Precision, (2) Sensitivity, (3) F1-Score, (4) Specificity and (5) Learning Rate and the above metrics computations are given below:

#### Precision

Precision [30] involves the discipline of the test in correctly predicting those who indeed have the disease out of all the people that the test said were affected. In other words, it indicates the extent to which the positive outcomes of the test indicated are due to lung cancer and are real positives, as shown in Eq. (13).

(13) $$Prec=\left(\frac{True_{Pos}}{True_{Pos}+False_{pos}}\right)\times \;100\%$$

#### Sensitivity or recall

Sensitivity [31] means the accuracy of a diagnostic test in the ability to classify individuals with lung cancer. It is defined as the proportion of individuals with lung cancer correctly identified by the test as shown in Eq. (14).

(14) $$Sensitivity=\left(\frac{True_{Pos}}{True_{Pos}+False_{neg}}\right)\times \;100\%$$

#### Specificity

Specificity [32] is an index of a diagnostic test that measures the capacity of the proposed model to identify patients without lung cancer as non−diseased. It defines the test's ability as separating those who do not have the disease from those who have lung cancer and minimizing false positives as shown in Eq. (15).

(15) $$Specificity=\left(\frac{True_{Neg}}{True_{Neg}+False_{Pos}}\right)\times \;100\%$$

#### F1-Score

F1-Score [33] gives an average of false positives and false negatives, more so in a situation where there are many more negative cases than positive ones. The harmonic means of precision and Recall integrates the two parameters in a single value. The computation of S1-Score is shown in Eq. (16)

(16) $$F1-Score=\frac{Prec*Rec}{Prec+Rec}$$

#### Learning rate

The learning rate [34] defines the number of steps the algorithm takes to reduce the error (or loss) during training. It determines the rate at which a machine learning model learns from the data or doesn't learn. As per the metrics computations discussed above, the data pre-processing was carried out using the Gabor Filter and Haar filter. The pre-processed data using the Gabor and Haar filters are fed into the proposed CNN architecture and the results are fetched. Also, the raw data is fed into the proposed CNN architecture and the results are fetched. Table 4 displays the results of the metrics of Gabor, Haar and non-pre-processed data.

**Table 4:** Comparison of results of non-pre-processed data, Haar and Gabor filter in CNN.

Metrics	Non-pre-processed with CNN	Haar Filter with CNN	Gabor Filter with CNN
Precision	50 %	75 %	97 %
Sensitivity (Recall)	100 %	83 %	97 %
F1-score	66 %	79 %	97 %
Specificity	0 %	73 %	98 %
Learning Rate	0.001	0.001000	0.001
Overall Training Accuracy	53.69 %	78.01 %	96.19 %
Overall Validation Accuracy	51.61 %	78.06 %	95.48 %

The Gabor achieved the overall best performance accuracy whereas, the Haar filter resulted in average performance accuracy and the non-pre-processed data showed very little performance. The Gabor filter scored high in all performance metrics; the range of precision, recall and F1-score is 97 %, and the specificity scored 98 % with a learning rate of 0.001. The training and validation accuracy for the Gabor filter is 96.19 % and 95.48 %, respectively. The Haar filter reached an average performance score in all the metrics, though the images are pre-processed. The precision, F1-score and specificity score were 75 %, 79 % and 73 % respectively and the sensitivity of the Haar filter was 83 % only. The overall training and validation accuracy of the Haar filter is only 78.01 % and 78.06 % respectively, which is relatively low compared to the Gabor filter. When the overall training and validation accuracy are compared for the Gabor and Haar filters, the Gabor filter has 18.18 % higher training accuracy and 17.42 % higher validation accuracy than the Haar filter. The non-pre-processed data are fed into the CNN model to check the biased part of the CNN. In the non-pre-processed data, the recall is 100 % as the system is identifying all the images as positive (i.e.) Malignant images. However, the specificity is zero because it means it is not able to calculate any negatives. This shows that CNN behaves as a very imbalanced model, such as overfitting or being a poorly balanced model for the raw data as input. As per the comparison in Table 4, the Gabor Filter with CNN achieves higher accuracies than the Haar filter and non-pre-processed data.

**Fig. 12:**
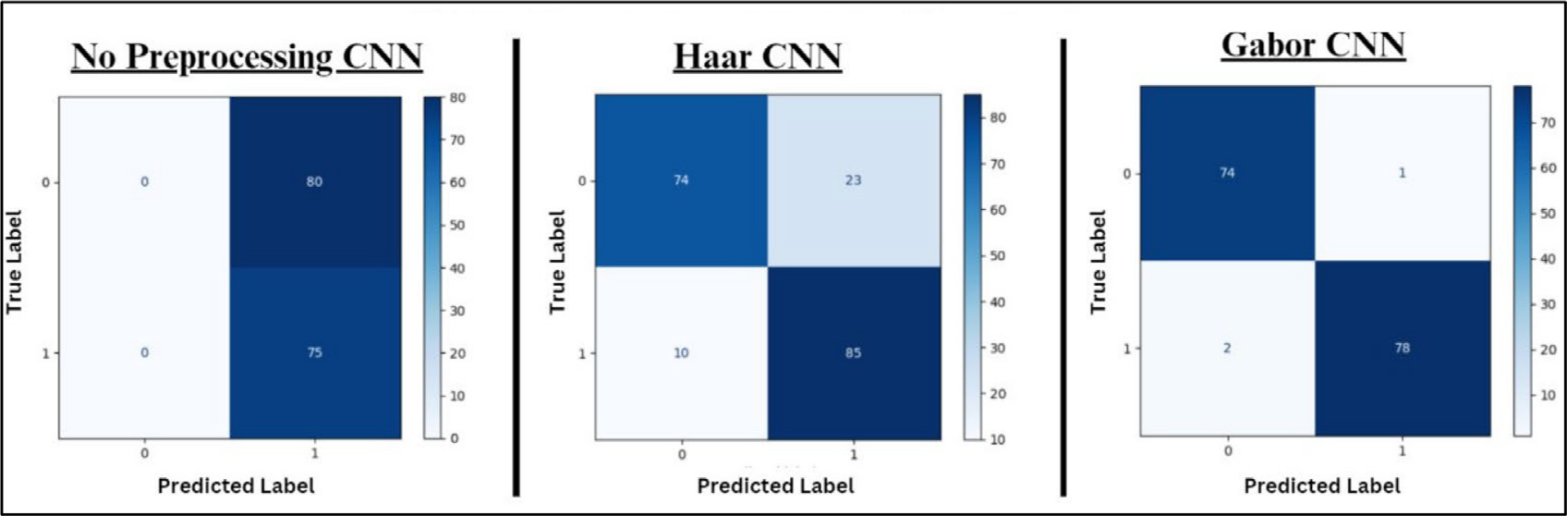
CNN confusion matrix comparison.

Fig. 12 depicts the CNN confusion matrix of 1) non-pre-processed data, 2) Haar Filter [35] and 3) Gabor Filter. In the confusion matrix of non-pre-processed data, though the true negative is high, the true positive value is zero, thus it fails to correctly predict the positive class (i.e. individuals who have lung cancer). In the Haar CNN, even though the value of true positive and true negative values is high which can predict whether the individual has lung cancer or not, the values of false positive and false negative also show reasonable values, resulting in incorrect predictions. Finally, Gabor CNN has good true positive and true negative with very minimal value in false positive and false negative. It shows that the Gabor CNN system has a balanced result, thus, the performance accuracy will be high. CNN confusion matrix comparison clearly illustrates that the Gabor filter has a high true positive and true negative and ensures accurate predictions.

Table 5 shows the comparison of results of non-pre-processed data, Haar and Gabor filter in the EMI-LTI model. Pre-processed data with Gabor filter, Haar filter and non-pre-processed data are fed as input into the EMI-LTI model and the performance metrics are captured. The overall training and validation accuracy of the non-pre-processed data is 52.01 % and 50.06 % respectively, which is very low. Also, the performance metrics, recall 100 % state that the system is identifying all the images as positive (i.e.) Malignant images. However, the specificity is zero because it means it is not able to calculate any negatives and clearly shows that non-pre-processed data with the EMI-LTI model is performing poorly with 50 % precision and 67 % F1-score. Haar filter with the EMI-LTI model performs in an average manner exhibiting overall training and validation accuracy of 78.8 % and 79.01 % respectively which is higher than the non-pre-processed data. The metrics precision, sensitivity, F1-score and specificity are 75.5 %, 81.3 %,77 % and 72.2 %, respectively. The score of the metrics achieved by the Haar filter represents the model exhibiting average accuracy.

**Table 5:** Comparison of results of non-pre-processed data, Haar and Gabor filter in EMI-LTI model.

Metrics	Non-pre-processed with EMI-LTI	Haar Filter with EMI-LTI	Gabor Filter with EMI-LTI
Precision	50 %	75.5 %	97.14 %
Sensitivity (Recall)	100 %	81.3 %	99.03 %
F1-score	67 %	77 %	98.08 %
Specificity	0 %	72.2 %	97.44 %
Learning Rate	0.001	0.001	0.001
Overall Training Accuracy	52.01 %	78.8 %	98.86 %
Overall Validation Accuracy	50.06 %	79.01 %	98.18 %

Finally, the Gabor filter shows greater accuracy when compared to the non-pre-processed data and the Haar filter. The Gabor filter showed overall training and validation accuracy of 98.86 % and 98.18 % respectively. The metrics precision, sensitivity, F1-score and specificity are 97.14 %, 99.03 %,98.08 % and 97.44 %, respectively. Not only did the Gabor filter show higher accuracy, but it also scored higher performance in all the metrics. As the paper suggested, the experiments show that the EMI-LTI model does better than CNN in all performance metrics and accuracies. The EMI-LTI model shows 2.67 % higher training accuracy than CNN and the EMI-LTI model shows 2.7 % higher validation accuracy than CNN.

**Fig. 13:**
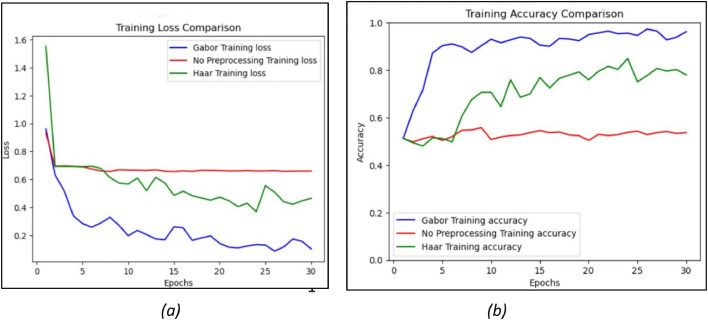
Comparison of CNN based on (a) training loss and (b) training accuracy.

The results of the CNN system of training loss [36] and training accuracy [37] are captured for all three systems: non-pre-processed, Haar filter and Gabor filter. Fig. 13 depicts the training loss and accuracy of the CNN model with non-pre-processed data, Haar and Gabor filter. To understand the performance of the machine learning models during training, capture the following: Training loss and Training accuracy metrics are needed.

Training loss is the implemented model's error on the training images. The training loss should be less to ensure that the model matches the training data well. However, it also shows that it might imply that it could lead to overfitting if the validation loss does not result in a similar trend. Training accuracy measures should predict how correctly the model predicts the trained images. It means the proportion of the model's correct classifications on the training data set. Validation loss is the error that the model makes using the validation. First, validation loss may slightly decrease with training loss. Sometimes, though, something like overfitting may occur; the validation loss will either not decrease or may even increase, while the training loss keeps on reducing. Validation accuracy indicates how correct the model's predictions are in relation to the validation data. It gives the proportion of the model's overall instances that get right on the validation data set. The training and validation accuracies do increase together although there is often a slight degradation in training accuracy when the model generalizes better to unseen data. However, if the validation accuracy decreases with increased training accuracy, the model is overfitting.

Fig. 13(a) compares the training loss of non-pre-processed Haar and Gabor filters. The training loss of the non-pre-processed data is high initially and gradually reduces at one point and becomes constant throughout the epochs thus, the training loss of the non-pre-processed data is high. In the Haar filter, though the training loss is lesser than in non-pre-processed data, the loss is higher than Gabor filter. However, we could observe that the Haar filter is consistent throughout the epochs. It clearly shows that the Gabor filter training loss is less when compared to Haar and non-pre-processed and Gabor training loss is also steady, showing that the system is not overfitting.

Fig. 13(b) compares the training accuracy of the non-pre-processed data, Haar filter and Gabor filter. The training accuracy of the non-pre-processed data is around 50 % throughout the epochs, indicating that the accuracy is much lower. As the training accuracy of the Haar filter is less initially, it slowly rises as the epochs increase. However, there are huge fluctuations in the training accuracy of the Haar filter as the epochs vary. It shows that the Haar filter is not consistent throughout the system. Compared to Haar and non-pre-processed, the CNN model with the Gabor filter has the highest training accuracy. The Gabor filter initially has very good training accuracy, increasing gradually as the epochs increase. In addition, there are not many variations and fluctuations in the accuracy as the epochs increase thus there is a consistent flow in training accuracy. The training accuracy of Gabor is 96.19 %, which indicates good accuracy.

**Fig. 14:**
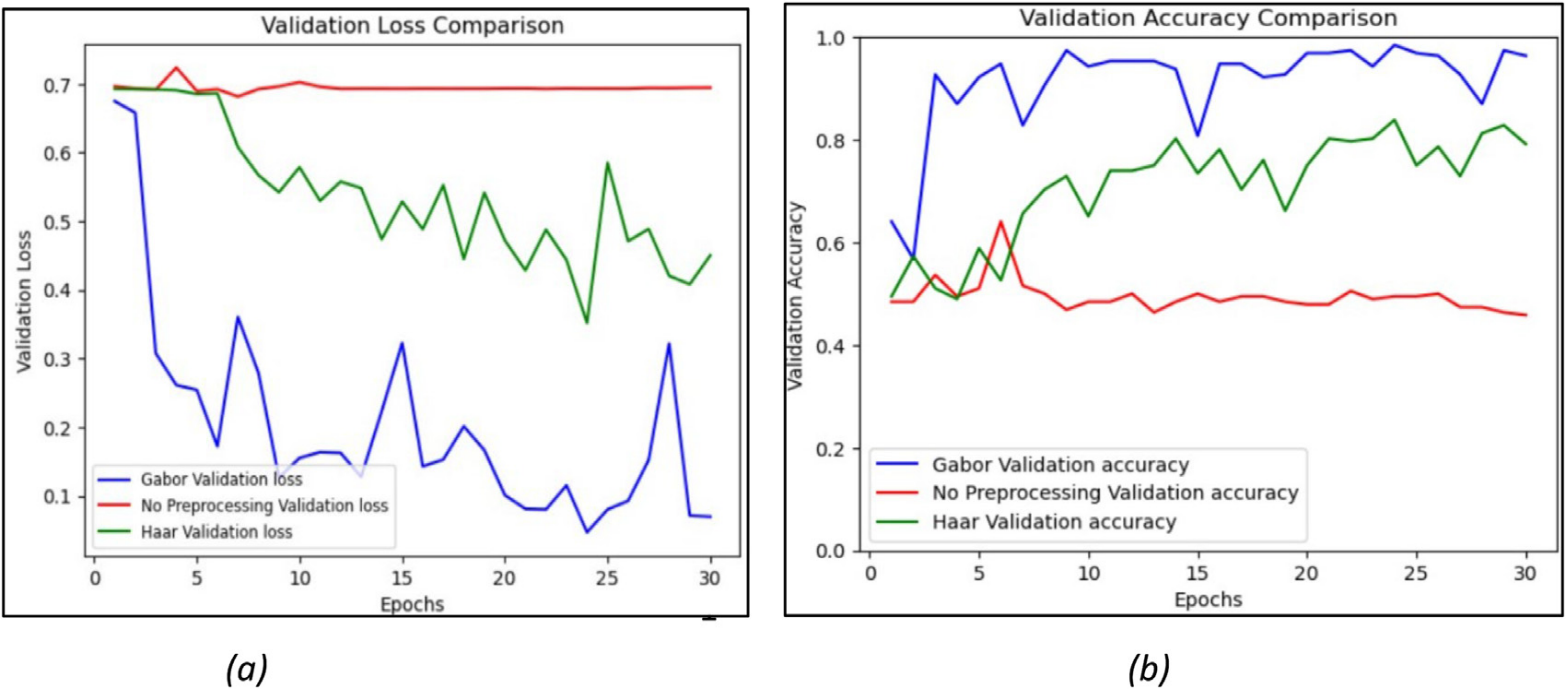
Comparison of CNN based on (a) validation loss and (b) validation accuracy.

Fig. 14(a) shows the comparison of the validation loss for non-pre-processed data, Haar filter and Gabor filter. The non-pre-processed data has around 70 % validation loss throughout the epochs, which is very high. The validation loss in the Haar filter is less than non-pre-processed data. The validation loss is negligible in the Gabor filter when compared to the Haar filter and non-preprocessing data. Though there are fluctuations in the Gabor filter validation loss, there is no continuous decrease in the training loss as shown in Fig. 13(a) thus, the model doesn't seem to be overfitting. Moreover, due to fluctuations, the CNN model learning rate does not vary as the learning rate of CNN with the Gabor filter is constant with a value of 0.001.

In Fig. 14(b), the validation accuracy of the non-pre-processed data is very low and it has become constant at one point. The validation accuracy of the Haar filter is higher than non-pre-processed data. However, the accuracy has not reached 80 % as the epochs vary. The validation accuracy of the Gabor filter has reached its maximum of 95.48 %. The validation and training accuracy in CNN are increasing, indicating that CNN is better at training unseen data. The validation accuracy is high compared to the Haar and non-pre-processing data.

**Fig. 15:**
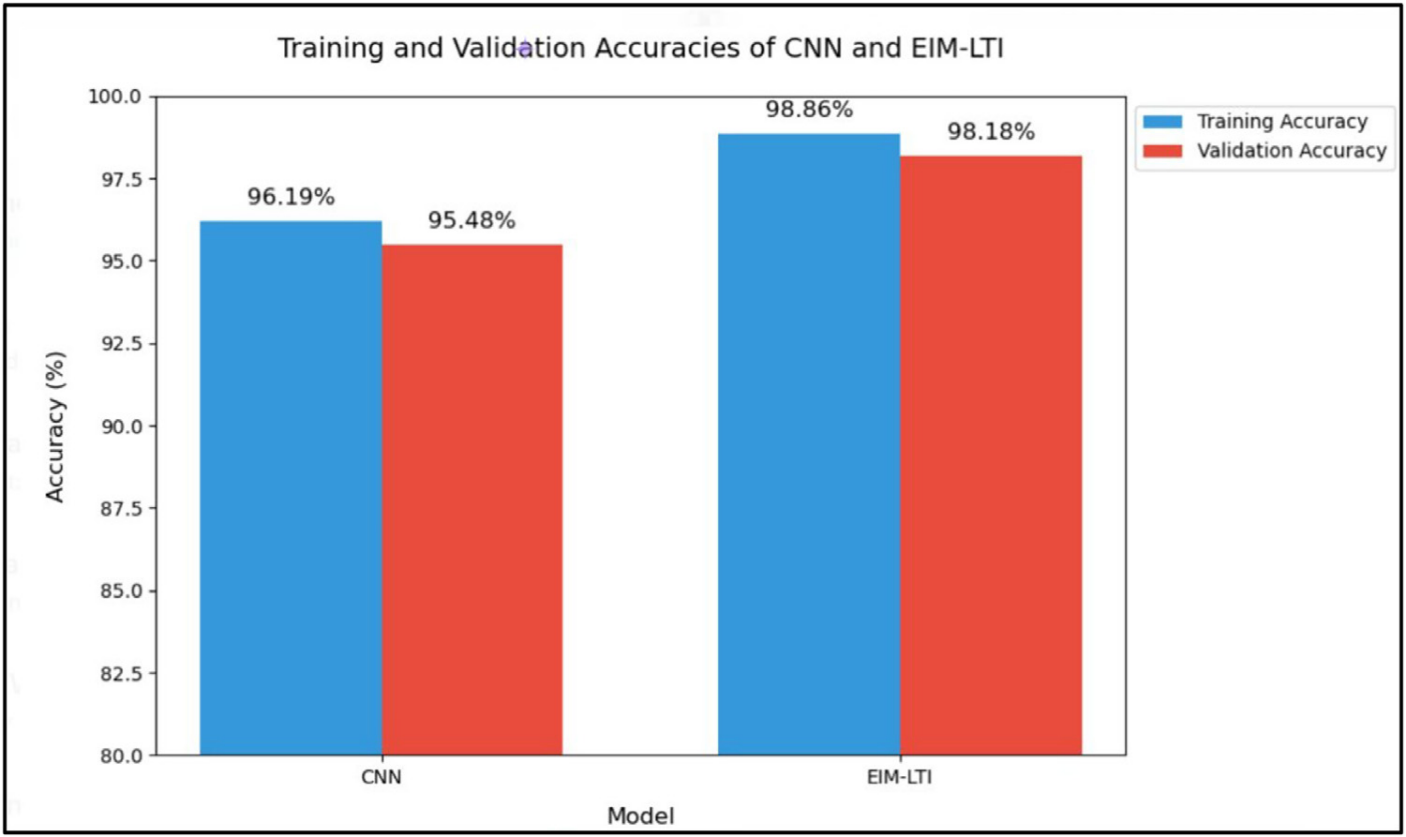
Comparison of training and validation accuracies.

Though CNN performs better in training and validation, the EIM-LTI model achieves greater training and validation accuracy than CNN. The training loss, accuracy, validation loss and accuracy for the EIM-LTI model cannot be depicted in Fig. 13, Fig. 14 because only the CNN model can run epochs but the SVM model cannot. Thus, the training and validation accuracy of the CNN and EIM-LTI models are depicted as bar charts in Fig. 15. CNN performs better in terms of losses; however, the training and validation accuracies are lesser than the EIM-LTI model. The main reason could be the lack of implementation of L2 regularization and dropout, which prevents overfitting. To overcome the overfitting in the EIM-LTI model, the L2 regularization and dropout are used and thus the accuracies are improved. The training accuracy of the EIM-LTI model is 2.67 % higher than the CNN and the validation accuracy of the EIM-LTI model is 2.7 % higher than the CNN as shown in Fig. 15. Thus, our proposed EIM-LTI model performed better than CNN.

**Fig. 16:**
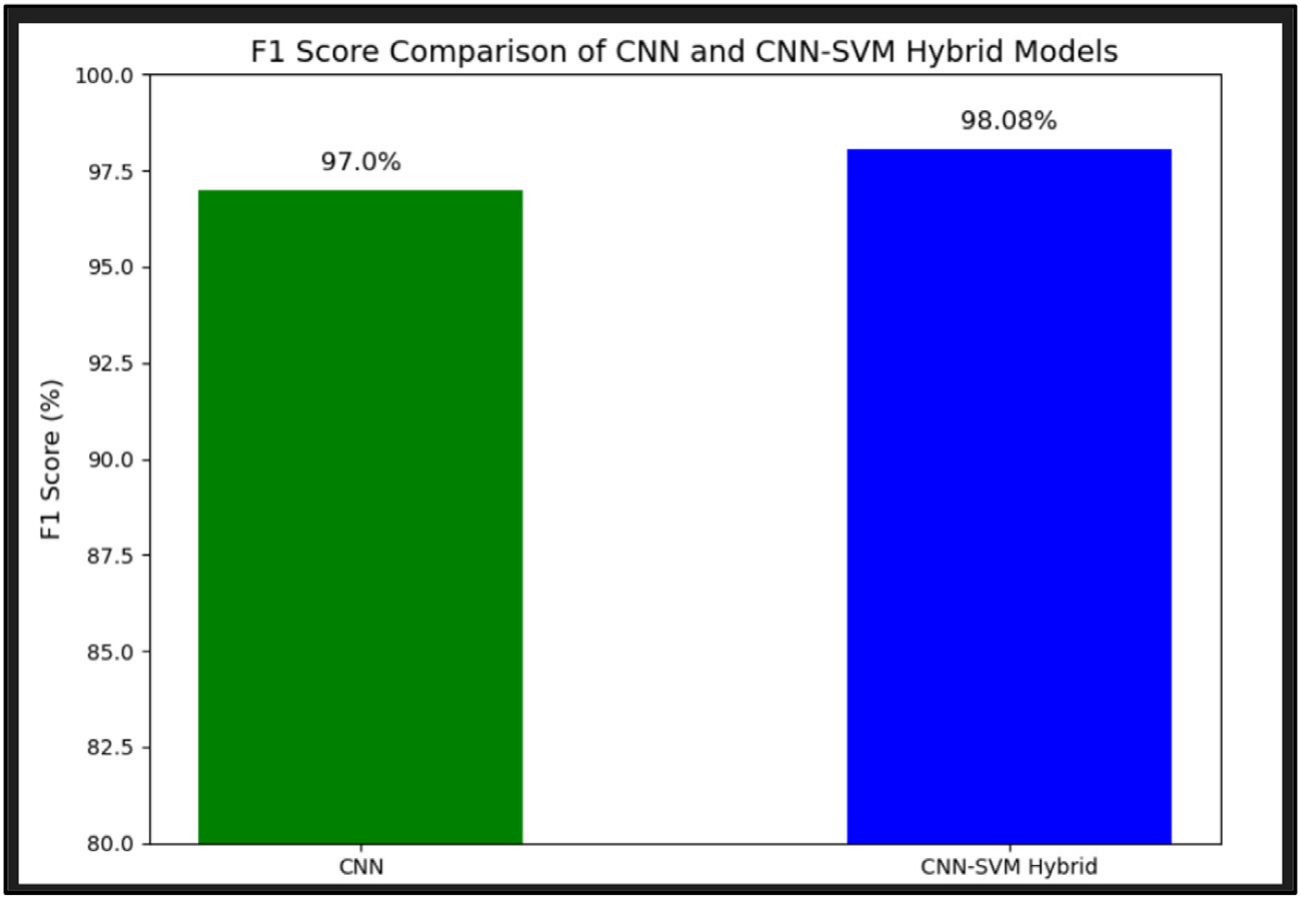
Comparison of F1-score.

The comparison of the F1-score is made between the CNN and the proposed EIM-LTI model in Fig. 16. A comparison of the F1 score is important as it can assess model performance by evaluating performance metrics such as precision and recall. The model should not be evaluated just by measuring the accuracy alone as it might mislead the results. The F1-score gives only one measure of how well the model is performing for the positives and at the same time ruling out the negatives, making it easier to compare which model is better at the end of the day. The F1-score is useful when comparing models to get an overall thorough and accurate measure of the performance of the models because it is reliable in situations where datasets are imbalanced and in most cases, where precision and recall both matters. Thus, among all the metrics, the F1-score is compared to evaluate the models, as the metric F1-score concentrates on the positive class and ensures a stable approach to managing class imbalance. The F1-score of EIM-LTI is high as SVM can adjust the class weights to enhance the performance of the minority classes. Thus, the hybrid model with CNN and SVM performs better. Fig. 16 depicts that the proposed model EIM-LTI has a higher F1-score than the CNN indicating that EIM-LTI has the highest possibility of accurate prediction of lung tumour.

**Fig. 17:**
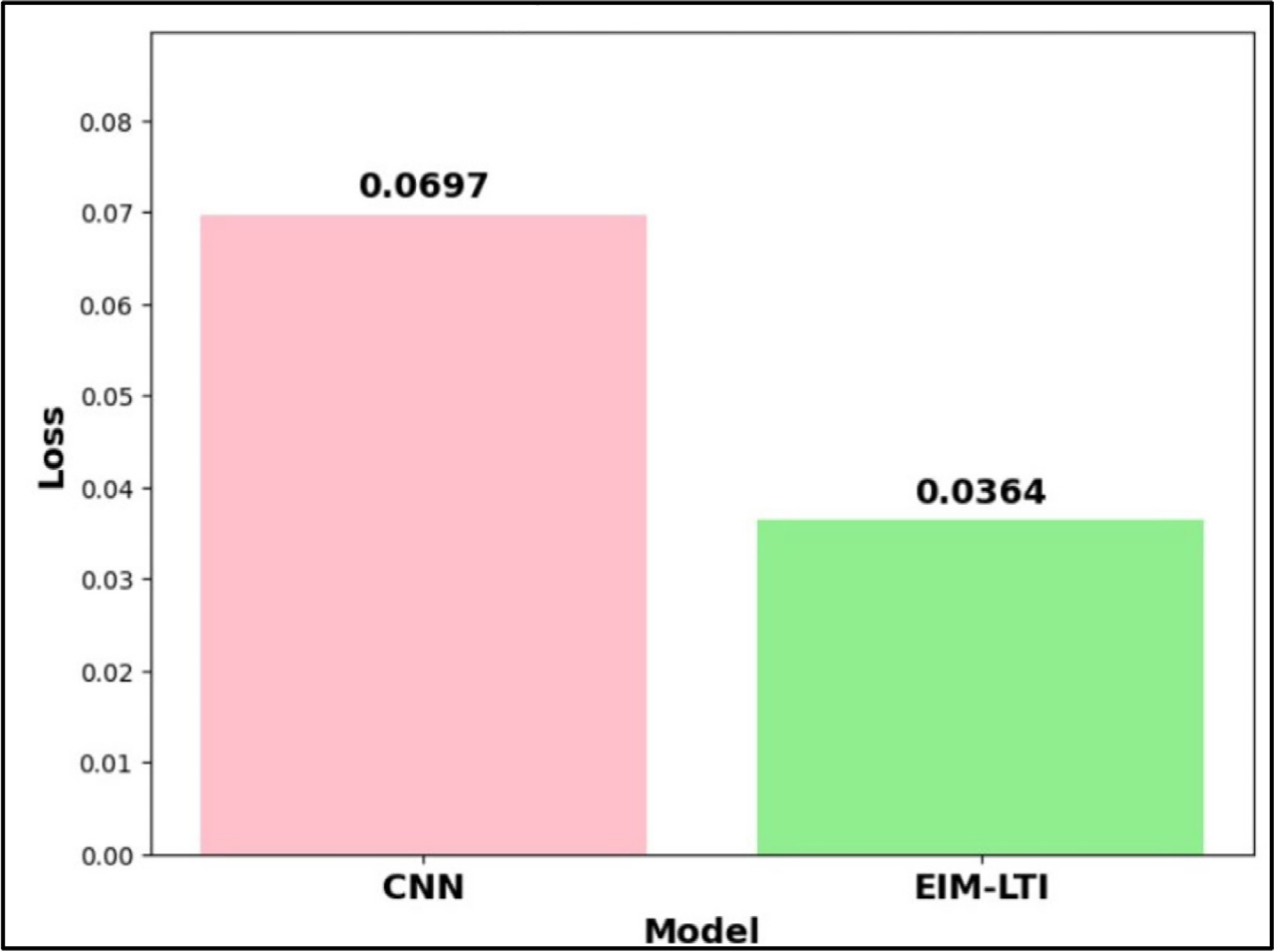
Loss comparison of CNN and EIM-LTI.

Fig. 17 depicts the comparison of loss between the models CNN and EMI-LTI. The comparison of validation loss provides various insights into the model. In this paper, two models were experimented and compared with three systems: non-pre-processed data, Haar filter and Gabor filter. As Gabor filter achieved more accuracies in both CNN and EIM-LTI models, Fig. 17, compares the models of Gabor filter with CNN and EIM-LTI. If the validation loss is high, then the problems of (1) model performance, (2) overfitting/underfitting, (3) model complexity and (4) learning rate need to be studied and analyzed. As per our experiment, both CNN and EIM-LTI models have lesser validation loss value of 0.0697 and 0.0364 respectively. When various models are compared, the one with the less validation loss will be generally ideal as it represents better performance. As shown in the above figure, the EIM-LTI model's validation loss is 0.0333 higher than CNN. Thus, we conclude that EIM-LTI model is a better model than CNN to take a broad view of unknown sample data and to avoid and diagnose the overfitting and underfitting.

##### Comparison of model accuracies for lung cancer detection

**Fig. 18:**
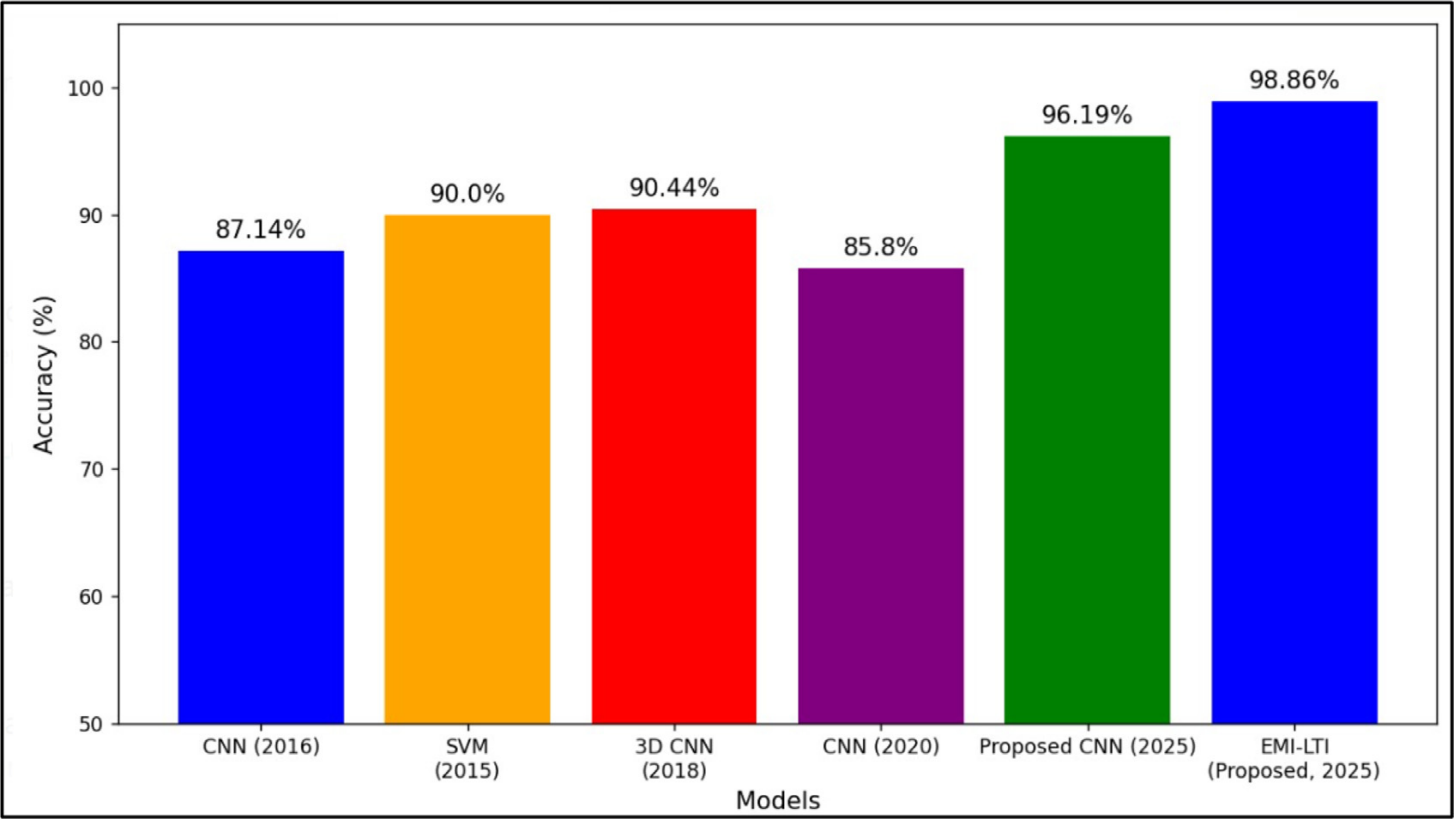
Comparison of model accuracies for lung cancer detection.

In this research paper, the accuracies of the two proposed models are compared with four existing models such as CNN (2016) [38], SVM (2015) [39], 3D CNN (2018) [40], CNN (2020) [41] as shown in Fig. 18. The accuracies of CNN (2016), SVM (2015), 3D CNN (2018) and CNN (2020) are 87.14 %, 90.0 %, 90.44 %,85.8 %, 96.19 % and 98.86 % respectively. Models established in recent years have achieved greater accuracy. In the graph, three CNN are compared, and the existing CNN (2016 & 2020) lingers behind in accuracy when compared to the proposed CNN (2025). The proposed CNN (2025) and EMI-LTI (2025) have achieved greater accuracy and are more consistent. Thus, the proposed CNN (2025) and EMI-LTI (2025) are reliable in cancer detection.

##### Cross validation

**Fig. 19:**
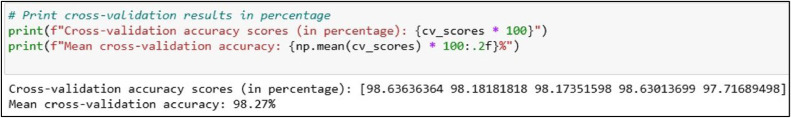
Code snippet of cross-validation performance accuracy.

**Fig. 20:**
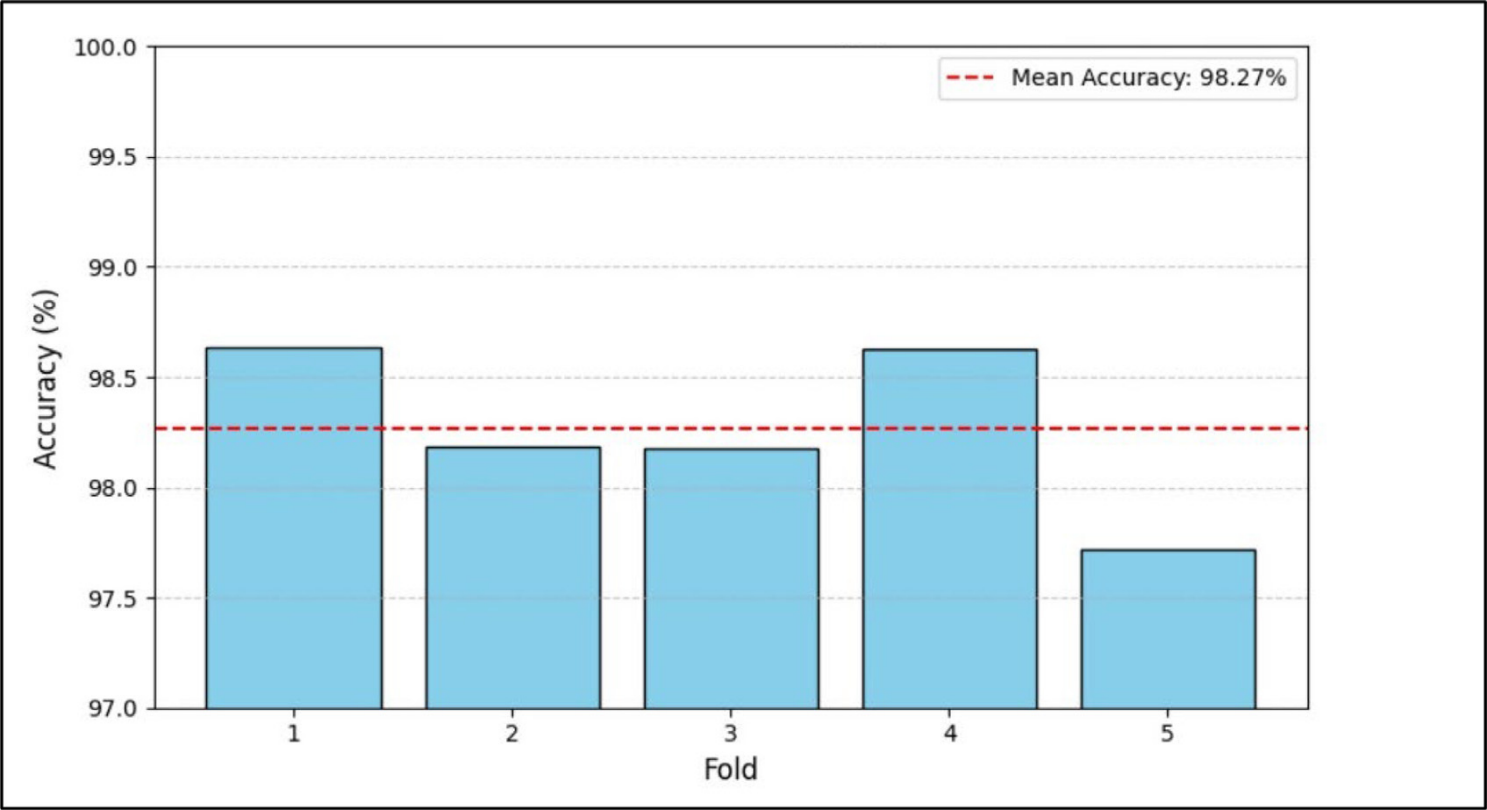
Cross-validation accuracy per fold.

Cross-validation is a broadly recognized methodology in machine learning. The technique uses multiple training-validation splits to produce a more reliable performance estimate of the model. Due to its iterative approach, the model can learn from various subgroups of data, eventually steering to a more general and steadier predictive performance. By using cross-validation approach organizations gain maximum data value and precise model performance evaluation through limited datasets.K-fold cross-validation technique has been employed to confirm the robust evaluation and generalization of the *EIM-LTI* model. During cross validation, the dataset is partitioned into five different folds. Among the five subsets of data, one of these was assigned as the validation set, while the other four subsets of data were applied for training the data in each iteration. The above process is repetitive iterative till every fold has been utilized as the validation set for once. The training and the validation sets are methodically varied, to mitigate the threat of overfitting and confirmed that the performance of the model was not inclined due to the data distribution. After the completion of five-fold cross validation, our model obtained an accuracy of 98.27 % through all the iterations. The achieved accuracy during its performance analysis proved that the model can effectively classify the data. Cross-validation serves as a performance metric and in hyperparameter tuning. The model is evaluated across various training-validation splits to adjust hyperparameters to improve predictive precision while inhibiting overfitting. Model reliability was proven by its reliable performance in all validation folds. Figs. 19 and 20 depict the cross-validation performance accuracy and the cross-validation accuracy per fold. The accuracy of five folds of cross-validation is 98.63 %, 98.18 %, 98.17 %, 98.63 % and 97.71 % respectively. Finally, the average accuracy is computed for five folds as 98.27 %. Thus, in Fig. 19, the code snippet of cross-validation performance accuracy is displayed that highlights the model's reliability and performance for various subgroups of the data. In Fig. 20, the graphical representation of the cross-validation accuracy results for five folds and the mean accuracy across all folds are depicted. Each blue bar in the graph indicates model accuracy for a precise fold. As per the experiment, fold 1 has the highest accuracy and fold 5 has the least accuracy. Mean accuracy provides a comprehensive report of model performance regardless of particular fold assessment.

#### Model evaluation on unseen data

To systematically measure the working of our EIM-LTI model, thorough testing on a new dataset that was not used in training or cross-validation practices was performed. This test on unseen data is essential to decide the generalization capability of the EIM-LTI model. During the model evaluation experimentation on a new dataset, an accuracy of 92 % was achieved. The accuracy achieved implies that the model is strong enough to generalize the unseen dataset. A decrease in the result of unseen data evaluation when compared to the training and validation process is probable due to the inherent variety and complications of real-world data, which might vary from the regulated circumstances of the training sets. As the new dataset was acquired, a sizable class imbalance was recognized. The newly formed dataset comprises of diverse categories of images leading to the unbalanced illustration of several classes. Data imbalance can adversely affect the performance of the model by biased predictions concerning the common classes, and thus the ability to identify and classify the marginal class samples is reduced.

**Fig. 21:**
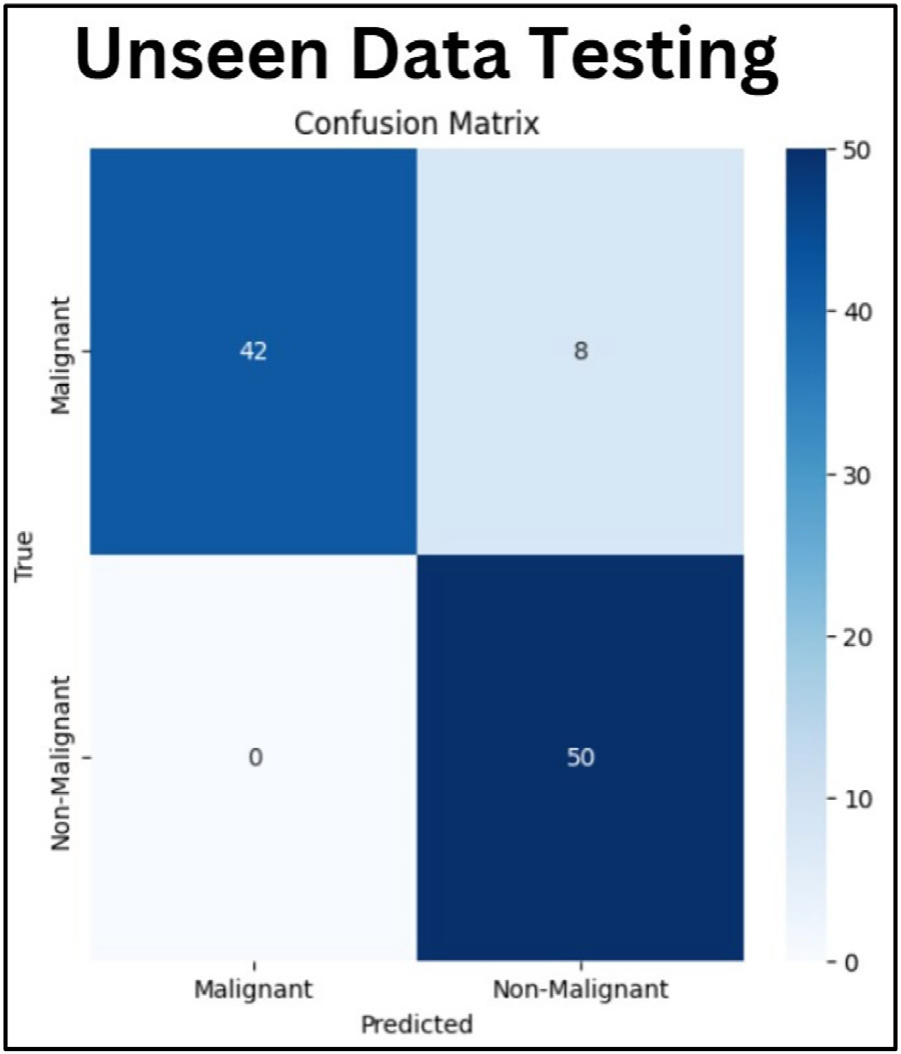
Confusion matrix.

To alleviate this issue, the dataset is curated by setting up a balanced dataset via selecting 50 non-malignant images and 50 malignant images. The images(50 non-malignant images and 50 malignant) are chosen for this study from the below link https://www.kaggle.com/datasets/mohamedhanyyy/chest-ctscan-images?resource=download. By selecting an equal number of images in both categories, an accurate estimation of the model's capability can be aided in recognizing among the category of normal and malignant cases. The process of dataset balancing prevented performance metrics from being influenced by the overrepresentation of any single class. Once the dataset balancing is done, the model is tested on the curated dataset. The model evaluation on the unseen dataset gave a refined comprehension of the model's ability to classify unknown data under balanced conditions. Using the balanced dataset helped us evaluate model sensitivity and specificity properly to prevent performance bias toward the majority class. Fig. 21 depicts the confusion matrix of the unseen data testing. Each cell displays the count of occurrences dropping into a certain category. The top left cell (true positive (TP), 42) displays the count of occurrences properly anticipated as malignant. The top right cell (false negative (FN), 8) displays the count of occurrences that are essentially malignant however they were anticipated as non-malignant. The bottom left cell (false positive (FP), 0) displays the count of occurrences that are essentially non-malignant, however they were wrongly anticipated as malignant. The bottom right cell (true negative (TN), 50) displays the count of occurrences accurately predicted as non-malignant. The model operates effectively on non-malignant occurrences with no FP, indicating it never incorrectly classifies a non-malignant occurrence as malignant. However, the model struggles a little with malignant occurrences as there are only 8 FN. These signify the lost findings of malignant occurrences, which are more needed in the current situation of the medical field.

**Fig. 22:**
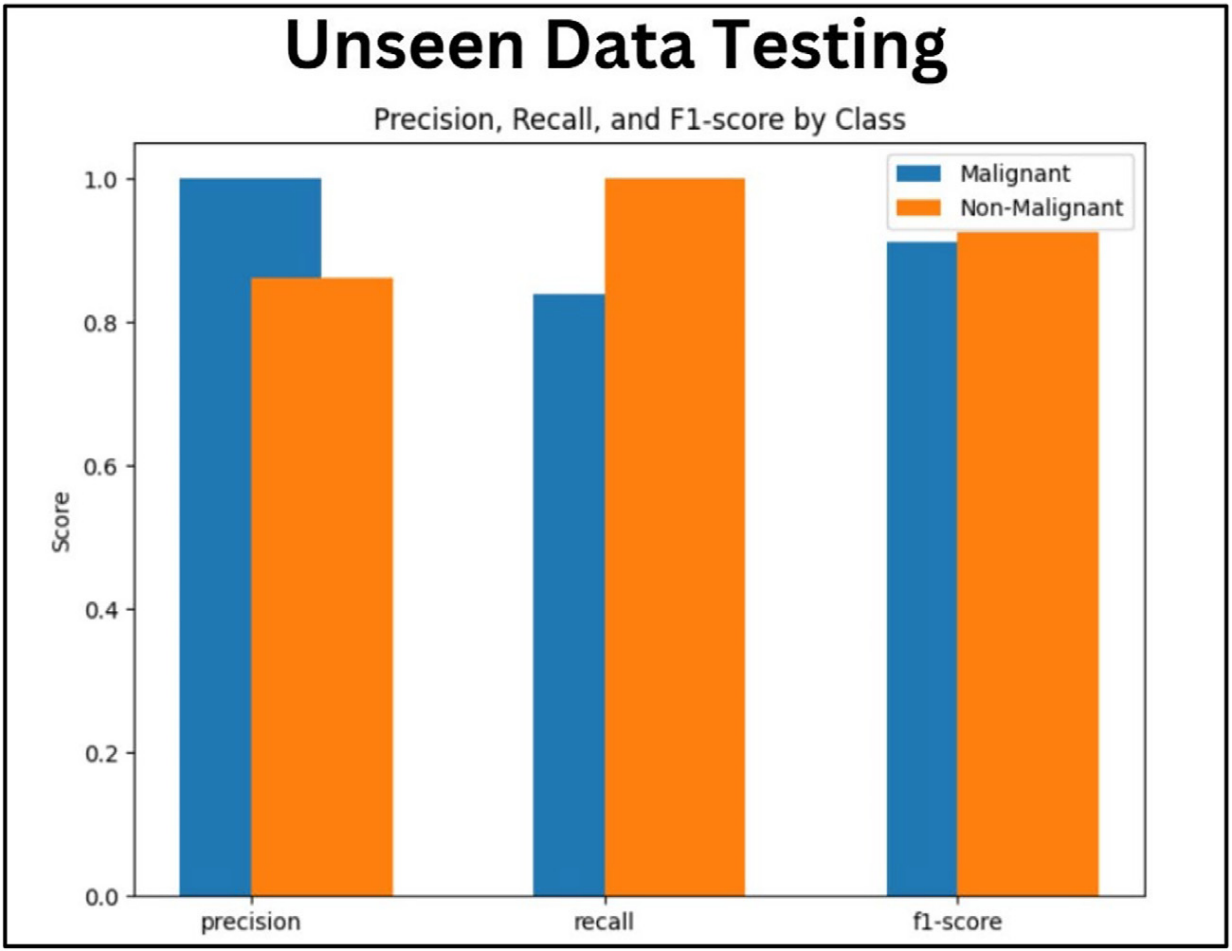
Comparison of the Precision, Recall and F1-Score.

Fig. 22 compares the results of Precision, Recall and F1-Score of unseen data for the two categories malignant and non-malignant. The precision result for the malignant class has an absolute precision of 1.0 stating that occurrences anticipated as malignant are correct. Recall measure resulted in 1.0 for the non-malignant class stating that all non-malignant occurrences are predicted correctly whereas recall for the malignant class is lesser (0.84) representing that 16 % of the correct malignant cases were not predicted correctly (FN). The F1-score of both malignant and non-malignant classes achieved a good score of 0.91 and approximately 0.98. As the F1-score is high and balances the precision and recall, the model represents more effective for both categories.

This research paper proposed two models, CNN and EIM-LTI, for lung tumor identification. Based on the experiments conducted, EIM-LTI model has a robust possibility of performing as a great potential method compared to CNN that results in the diagnosis of lung cancer. The EIM-LTI model reported in the study presents improved precision, sensitivity and specificity for discriminating malignant and non-malignant CT image scans. This study performed experiments using CNN and the EIM-LTI model with non-pre-processed data, Haar and Gabor Filter and comparisons between non-pre-processed data, Haar and Gabor Filter show the media-robustness of the EIM-LTI model, which again yields better results compared to traditional CNN models. The proposed EIM-LTI achieved 98.86 % and 98.18 % of training and validation accuracy, respectively higher than CNN and the validation loss of the EIM-LTI model is 0.0333 higher than CNN. While both the CNN and the EIM-LTI models may offer high accuracy, this study has found that the EIM-LTI model architecture may even surpass the independent CNN model in terms of high accuracy and insensitivity to data dimensionality. Hence, using EIM-LTI, there are a wide variety of opportunities for providing highly precise and initially diagnosed lung cancer and helping medical practitioners make more proper decisions and improve treatment results. In this study, the performance accuracy of the two proposed models is compared with four existing models such as CNN (2016), SVM (2015), 3D CNN (2018) and CNN (2020) proved that our proposed models had performed better. Cross-validation is implemented on the proposed EIM-LTI model by partitioning the dataset into 5-folds obtaining an accuracy of 98.27 % through all the iterations. The resulting cross-validation accuracy proved that the model can effectively classify the data. Finally, the working of the EIM-LTI model is systematically measured via the evaluation of unseen data and the results are measured through metrics such as Precision, Recall and F1-Score. The metric results proved the model's effectiveness and reliability in classifying the malignant and non-malignant image occurrences correctly. As a future perspective, the proposed CNN and EIM-LTI need to be applied to larger and more diverse datasets and the models should be extended such that they can diagnose different types of cancers to demonstrate their adaptability in medical imaging.

## Limitations

The main limitation of the EIM-LTI model could be its more computational resources and time when compared to the CNN as the EIM-LTI model needs various additional steps concerned in feature extraction and classification. The model's precision and recall for malignant occurrences need to be enhanced such that the significant cases can be more concentrated.

## Ethics statements

This research does not involve human subjects.

## Declaration of competing interest

The authors declare that they have no known competing financial interests or personal relationships that could have appeared to influence the work reported in this paper.

## Data Availability

Data will be made available on request.
